# Amperometric Biosensor for Quantitative Measurement Using Sandwich Immunoassays

**DOI:** 10.3390/bios13050519

**Published:** 2023-05-05

**Authors:** Thor Pedersen, Peter Fojan, Anne Kathrine Nissen Pedersen, Nils E. Magnusson, Leonid Gurevich

**Affiliations:** 1 Department of Materials and Production, Aalborg University, Skjernvej 4A, 9220 Aalborg, Denmark; tp@biostrip.dk (T.P.); fp@mp.aau.dk (P.F.); 2 Biostrip APS, Lindevangsvej 10, 8240 Risskov, Denmark; 3 Medical Research Laboratory, Department of Endocrinology and Internal Medicine, Aarhus University Hospital, Palle Juul-Jensens Boulevard 165, 8200 Aarhus, Denmark; aknp@clin.au.dk

**Keywords:** biosensor, immunoassay, surface characteristics, electrochemical sensing, screen printed electrode

## Abstract

State-of-the-art clinical detection methods typically involve standard immunoassay methods, requiring specialized equipment and trained personnel. This impedes their use in the Point-of-Care (PoC) environment, where ease of operation, portability, and cost efficiency are prioritized. Small, robust electrochemical biosensors provide a means with which to analyze biomarkers in biological fluids in PoC environments. Optimized sensing surfaces, immobilization strategies, and efficient reporter systems are key to improving biosensor detection systems. The signal transduction and general performance of electrochemical sensors are determined by surface properties that link the sensing element to the biological sample. We analyzed the surface characteristics of screen-printed and thin-film electrodes using scanning electron microscopy and atomic force microscopy. An enzyme-linked immunosorbent assay (ELISA) was adapted for use in an electrochemical sensor. The robustness and reproducibility of the developed electrochemical immunosensor were investigated by detecting Neutrophil Gelatinase-Associated Lipocalin (NGAL) in urine. The sensor showed a detection limit of 1 ng/mL, a linear range of 3.5–80 ng/mL, and a CV% of 8%. The results demonstrate that the developed platform technology is suitable for immunoassay-based sensors on either screen-printed or thin-film gold electrodes.

## 1. Introduction

Over the past decades, the detection of biomarkers has gained increasing attention. The timely detection and quantification of proteins, nucleic acids, or small molecules characteristic of a particular medical condition or related to a specific infection or contamination agent are of great importance in a variety of fields, including medicine, food safety, drug discovery, and quality and environmental control [1,2,3]. A common way to selectively detect a specific biomarker involves immunoassays, where target recognition molecules such as antibodies or aptamers are employed due to their high specificity, affinity, and stability. Typically, sandwich immunoassays are used, where a target analyte is sandwiched between surface-immobilized capture (primary) antibodies and labeled secondary antibodies. The formation of these complexes enables the quantification of the analyte concentration utilizing the label on the secondary antibody. Most commonly, the label is involved in a reaction that generates an optical signal which is proportional to the analyte concentration. The gold standard is ELISA, where the label is an enzyme, e.g., horseradish peroxidase (HRP). In the presence of hydrogen peroxide, HRP oxidizes a cofactor, producing a colored or fluorescent product for optical quantification [4]. However, these methods are time-consuming and require specialized equipment and trained personnel, which makes them unsuitable for use outside of laboratory settings.

Combining sandwich immunoassay with electrochemical detection is especially attractive due to its inherent advantages such as its low cost, miniaturization, simplicity, high sensitivity, customization, and ease of operation, leading to sensors that can be used outside of laboratories [5,6,7,8,9]. The most common electrochemical detection schemes employ one of the four following approaches: (1) labelling with a redox enzyme, (2) electrochemical impedance spectroscopy (EIS), (3) labelling with nanoparticles, and (4) magnetoimmunosensing, among which the first two methods account for the majority of studies [7,10]. In the first case, an enzymatic label, typically HRP, generates electroactive species that can be detected electrochemically using cyclic or pulse voltammetry or amperometry. An important advantage of this approach is that existing ELISA assays can be transferred to an electrochemical platform, assuming that a suitable electroactive substrate and detection technique are in place. Furthermore, HRP, as a label, offers multiple advantages due its robustness, relatively small size, inexpensiveness, high turnover rate, and compatibility with a large variety of electroactive substrates [11,12,13,14,15]. On the other hand, EIS sensors can operate label-free and involve an external redox probe, usually ferro/ferricyanide or ferrocene, present in the solution. The signal stems from the change in the charge transfer resistance and double-layer capacitance due to the formation of immunocomplexes on the electrode surface [16]. There is a growing demand for tools that enable diagnosis and monitoring in PoC environments, with applications in the fields of personalized medicine for the monitoring of treatment and disease protein biomarkers, as well as early diagnosis, e.g., of COVID-19 [17,18]. To facilitate the translation of PoC into clinical practice and home-care in the future, rapid, robust, and cost-efficient systems are required.

The NGAL assay was used as a model system for the biosensor. The early detection of NGAL is of importance for identifying acute kidney injury (AKI). The NGAL concentration increases within 2 h and can be indicative of AKI up to 48 h prior to a clinical diagnosis [19,20]. In healthy individuals, the concentration of NGAL is generally below 50 ng/mL, depending on the assay, while upon acute kidney injury, it rises significantly [20,21]. The main objective of the present study was to develop an immunoassay-based electrochemical biosensor platform suitable for a range of biomarkers for applications in PoC environments.

## 2. Materials and Methods

### 2.1. Materials

N-hydroxysuccinimide (NHS, 98%), monopotassium phosphate, potassium chloride, TWEEN^®^20, bovine serum albumin (BSA, ≥96%), 11-mercaptpundocanoic acid (MUA, 95%), N-(3-dimethylaminopropyl)-N’-ethylcarbodiimide hydrochlorid (EDC, ≥99%), polyvinyl alcohol (PVA), cysteamine (Cyst, 95%), 2-hydroxy-4′-(2-hydroxyethoxy)-2-methylpropiophenone (Irgacure, 98%), polyethylene glycol diacrylate with a M_w_ = 575 (PEGDA), trizma^®^ base, potassium hexacyanoferrate(II) trihydrate, potassium hexacyanoferrate(III), and 2-(N-Morpholino)ethanesulfonic acid (MES, ≥99%) were obtained from Sigma Aldrich. Sulfuric acid, sodium chloride, disodium phosphate, and absolute ethanol were obtained from VWR, and 1-Step Turbo TMB-ELISA Substrate Solution was obtained from Thermo Scientific.

Milli-Q water was used for all aqueous solutions. Phosphate buffer saline (PBS) with a pH of 7.3 was prepared using 137 mM sodium chloride, 2.7 mM potassium chloride, 10 mM disodium phosphate, and 1.76 mM monopotassium phosphate. Screen-printed electrodes (SPEs) were acquired from Metrohm DropSens, Germany (models 220BT and 220AT) and PalmSens, Netherlands (model ItalSens Gold), while the thin-film interdigitated electrodes (IDE) were from Metrohm DropSens, Germany (model G-IDEAU10) and MicruX, Spain (model ED-IDE1-Au).

Immunoassays: Human low-density lipoprotein (LDL) was obtained from Sigma Aldrich. Anti-human apolipoprotein B monoclonal antibodies (LDL20|17) and biotin-conjugated anti-human apolipoprotein B monoclonal antibody (LDL11) were supplied by MABTECH. Anti-human Lipocalin-2 monoclonal antibody (MAB17571), biotin-conjugated anti-human Lipocalin-2 polyclonal antibody (BAF1757), recombinant human Lipocalin-2, anti-human VEGF165 polyclonal antibody (AF293), biotin-conjugated anti-human VEGF165 (BAF293), recombinant human VEGF165, and streptavidin-conjugated horseradish peroxidase (Strep-HRP) were from R&D systems. The NGAL assay was previously validated [22].

### 2.2. Chemiluminescence

To verify the functionalization of antibodies to the working electrode, sandwich assays were conducted or surface-bound antibodies were detected using chemiluminescence. A standard Western blotting procedure was applied. In brief, functionalized electrodes were blocked with 2% BSA followed by incubation with species-specific HRP-conjugated antibodies for the direct detection of the functionalized antibody or incubation with antigen and HRP-conjugated detection antibodies (for assay detection). The reaction volumes were 5–20 µL depending on the working electrode areas. An incubation time of 1 h was used for every step, and the electrodes were washed four times in PBS with 0.1% TWEEN20 (PBS-T) followed by washing four times in PBS between each step. The electrodes were dried before applying the substrate, Clarity Western ECL Substrate from BioRad. The signal was read using a ChemiDoc Imaging System from BioRad.

### 2.3. Preparation of Anitbody–Gold Nanoparticle Conjugates

Gold nanoparticles (AuNPs) were synthesized by preparing 100 mL of 1 mM HAuCl4 in a two-neck, round-bottom flask and titrated to pH 4 with 200 µL of 1 M sodium hydroxide. The flask was then placed on a heating plate equipped with a condenser and heated while stirring. After reaching the boiling point, 10 mL of 38.8 mM sodium citrate was added, and a dark blue color was obtained after 1 min. The solution was then boiled for 15 min. The AuNP solution was allowed to cool down to room temperature (RT) and then transferred to a glass bottle for storage and left overnight.

The AuNP solution was then used to create BAF1757-AuNP conjugates via passive adsorption. In short, 17 µg of BAF1757 was diluted in 50 mM TRIS buffer of pH 7.5 before the addition of 1 mL AuNPs and then vortexed for 5–10 s. The solution was left for 30 min at RT before adding PBS-T and 0.1% BSA. The unbound antibody was removed via two consecutive centrifugations, in which the supernatant was removed. The pellet was dissolved and stored at 4 °C.

### 2.4. Custom Thin-Film Electrode Fabrication

The electrodes were fabricated using standard optical lithography techniques. Working and counter electrodes were prepared via E-beam evaporation of 150 nm of gold with 5 nm of chromium underneath. The reference electrode was prepared using E-beam evaporation of 200 nm of silver with a 5 nm chromium underlayer. Before usage, the silver reference was electrochemically chloridized by applying a current density of 1 mA/cm^2^ for 60 s in a 1 M potassium chloride solution using the on-chip working electrode as a counter and an external Ag/AgCl electrode as a reference [23].

### 2.5. Immunosensor Functionalization

#### 2.5.1. Self-Assembled Monolayer (SAM) Formation

Before forming the SAM of MUA, all the electrodes were cleaned to remove impurities. The electrode surface was cleaned using cyclic voltammetry (CV) in a 50 mM solution of H_2_SO_4_. The solution was added to the electrode and subjected to 10 cycles of CV from −0.1 V to 1.4 V with a scan rate of 100 mV/s and a step size of 2 mV.

The formation was carried out following standard protocols, as outlined in [24,25,26,27]. In short, MUA was dissolved in EtOH and then diluted with Milli-Q water. The electrodes were immersed in the solution overnight at 4 °C and thoroughly rinsed afterwards using absolute ethanol and Milli-Q water to remove the physisorbed MUA.

#### 2.5.2. Antibody Immobilization

The immobilization of the antibody on the SAM layer was performed using EDC/NHS coupling, forming a covalent bond between the carboxylic acid and a primary amine, as previously described [24,28,29]. The electrodes were exposed to a fresh 1:2 solution of NHS and EDC in a 50 mM MES buffer at pH 5.0 for 40 min and subsequently rinsed. Immediately after, a 5 µg/mL antibody solution was pipetted onto the electrode and left for 1 h at RT. Then, the electrodes were rinsed to remove excess antibody and dried with a nitrogen flow in preparation for the blocking procedure.

#### 2.5.3. Blocking of Electrode Surface

A blocking step is crucial for preventing nonspecific interactions of antigen, antibodies, or other molecules present in complex samples with the electrode surface. A solution of 1.5% PVA was pipetted onto the electrodes and left overnight at 4 °C. After the overnight step, the electrodes were rinsed with PBS.

A different blocking method was employed for the urine samples. A solution of 1 mM cysteamine in PBS was pipetted onto the electrodes and incubated overnight at 4 °C, quenching the EDC/NHS groups that did not react with the antibody and filling in the MUA-free gaps on the electrode surface. After the overnight step, the electrodes were rinsed with PBS and gently dried using a nitrogen flow. To complete the blocking, the electrodes were exposed to a fresh solution of 1% PEGDA and 0.05% Irgacure in PBS. First, Irgacure was completely dissolved in PBS by heating the solution to 40 °C for 40 min before adding PEGDA. The solution was pipetted onto the electrodes and exposed to UV light for 2 min. After exposure, the electrodes were thoroughly washed with PBS-T and PBS.

### 2.6. Assay Procedures

The assays below were performed by (i) preparing the samples; (ii) transferring 15 uL to the immunosensor and incubating for 30 min at RT while covered to form the bond between the biotin and streptavidin; (iii) thoroughly washing the sensors using PBS-T and/or PBS; and (iv) measuring the immunosensors.

#### 2.6.1. Strep-HRP Assay

The samples were prepared as a dilution of Strep-HRP in PBS-T with 0.1% BSA and were measured via chronoamperometry.

#### 2.6.2. NGAL Assay for CV and EIS

The samples were prepared as a dilution series using PBS-T with 0.1% BSA, and each immunosensor was incubated with every concentration, starting from the lowest.

#### 2.6.3. Assay for Chronoamperometry

The AuNP conjugate solution was diluted 1:60 using a solution of Strep-HRP diluted 1:50 in PBS-T. The samples were diluted using PBS with 0.2% TWEEN20 and 0.2% BSA and then mixed in a 1:1 ratio with the prepared conjugated solution and pre-incubated for 30 min at RT before application to the sensor.

The assay for VEGF165 and LDL followed the same procedure but with the detection antibody at 0.4 µg/mL that was diluted using a solution of Strep-HRP instead of the conjugate solution.

The urine samples for the NGAL assay were prepared by pooling control urine samples from healthy volunteers to form a comprehensive sample matrix for assay optimization. The urine pool was aliquoted, snap-frozen, and stored at −80 °C until usage. Testing solutions were prepared in a 1:4 diluted urine pool with PBS containing 0.2% TWEEN20 and 0.2% BSA.

### 2.7. Electrochemical Measurements

All electrochemical measurements were performed with an on-chip, three-electrode setup consisting of a gold working and counter electrode and an internal Ag/AgCl reference electrode using an Anapot EIS potentiostat (Zimmer & Peacock, Horten, Norway) or Sensit Smart potentiostat (PlamSens, Houten, The Netherlands). Unless otherwise specified, the electrode used for the immunosensors was 220BT. All potentials are specified with respect to the internal Ag/AgCl reference electrode.

Electrochemical characterization was performed with CV and EIS measurements using 3 mM ferro/ferricyanide in PBS. CV was performed with 5 cycles using a potential window ranging from −0.3 V to 0.6 V, with a scan rate of 100 mV/s and a step size of 2 mV. EIS was performed from 100 kHz to 0.1 Hz with a DC potential of 120 mV and an AC excitation of 10 mV.

For the immunoassay testing, CV, EIS, and chronoamperometry were used. CV and EIS were performed using 3 mM ferrocenedimethanol in PBS with the same settings as those used in characterization, except for the DC potential and AC excitation, being 170 mV and 5 mV, respectively. Chronoamperometry was performed with the 1-Step Turbo TMB-ELISA Substrate Solution for 120 s with a fixed potential at 0.0 V.

## 3. Results and Discussion

### 3.1. Electrode Surface Characterization

**Figure 1:**
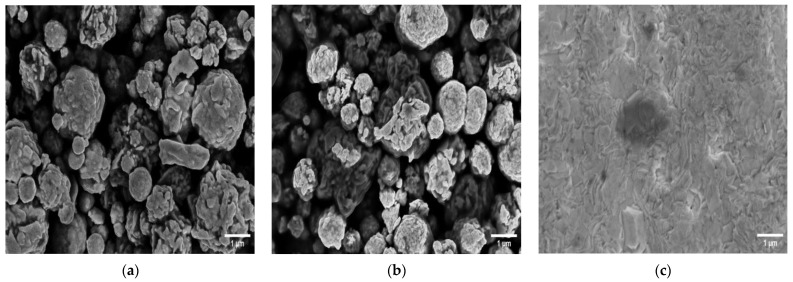

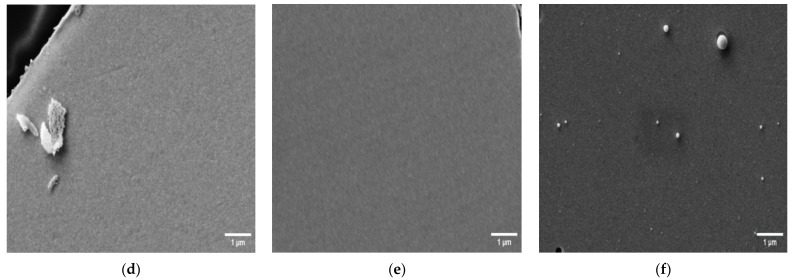
SEM images of (**a**) 220BT SPE; (**b**) ItalSens SPE; (**c**) 220AT SPE; (**d**) MicruX IDE; (**e**) Metrohm IDE; (**f**) custom-made electrode at 13k magnification.

**Figure 2:**
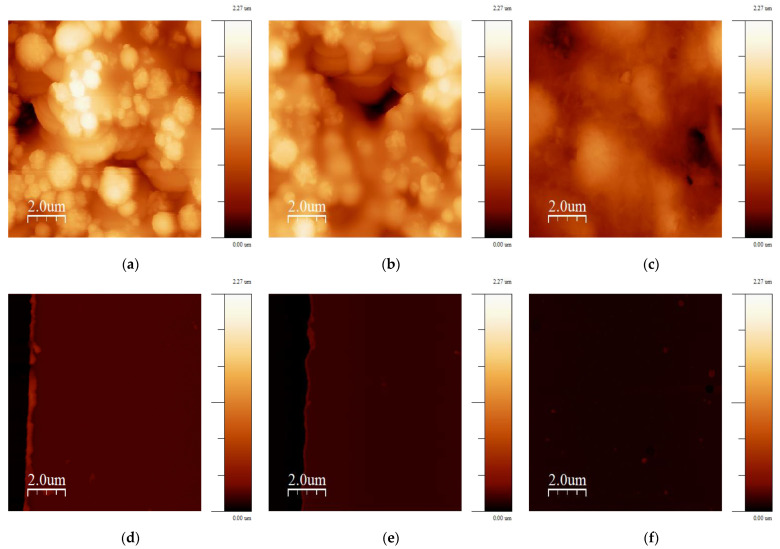
10 × 10 µm AFM images of (**a**) 220BT; (**b**) ItalSens; (**c**) 220AT; (**d**) MicruX IDE; (**e**) Metrohm IDE; (**f**) custom-made electrode. The vertical scale is the same for all AFM images.

The morphology of several SPEs and thin-film electrodes, which have been used previously by other groups for biosensor applications, were characterized in this work using scanning electron microscopy (SEM) and atomic force microscopy (AFM) [30,31,32,33,34,35,36,37,38,39,40,41,42]. Figure 1 shows SEM images of the electrodes at 13k magnification, and Figure 2 shows 10 × 10 µm AFM images. The 220BT and ItalSens electrodes exhibit a granular morphology, as seen in Figure 1a,b and Figure 2a,b, with nonuniform grains protruding outward and significant variation in height. The 220AT electrodes show a partially smoothed morphology composed of fused flakes, as seen in Figure 1c and Figure 2c. All the thin-film electrodes, further referred to as MicruX, Metrohm and custom-made, have smooth surfaces with a roughness on the nm scale, as seen in Figure 1d–f and Figure 2d–f.

**Figure 3:**
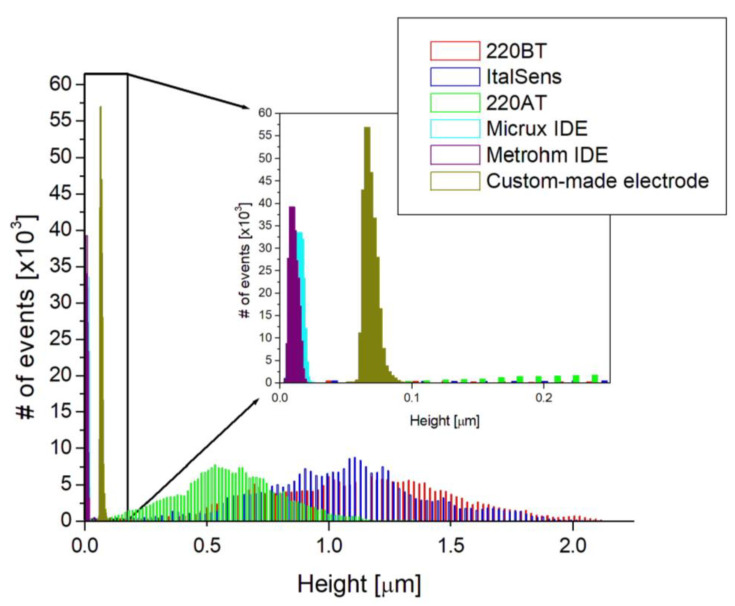
Height histograms based on AFM images shown in Figure 2. Insert shows a zoom-in of the height histogram.

The AFM images were used to further investigate the morphologies by estimating the RMS surface roughness, calculating the roughness coefficient, defined as the ratio of the ironed surface area to the scan area, and constructing a histogram of heights. Table 1 presents the values obtained from the surface roughness analysis, and Figure 3 shows the histogram. The RMS roughness is 50–100 times higher for the SPEs compared to the thin-film electrodes, indicating a significantly rougher surface with a broad height distribution, as shown in Figure 3. This is also supported by the roughness coefficient values, since they have an increase of 11–69% for the SPEs and are within 3% for the thin-film electrodes. This means that the actual surface area of the SPEs is substantially larger than the scan area due to the rough topography, whereas the thin-film electrodes, with their flat topography, have a negligible increase.

### 3.2. Electrochemical Characterization of Immunosensor

**Figure 4:**
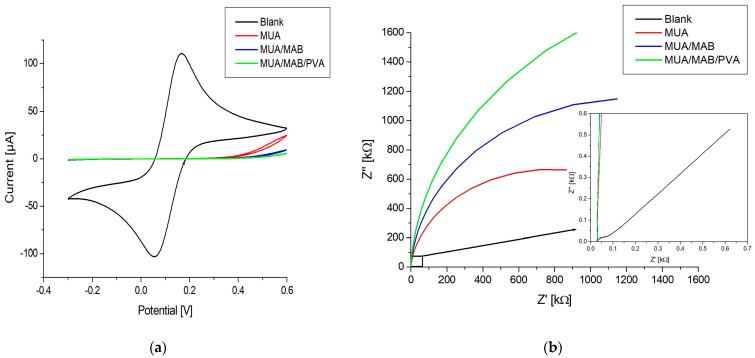
(**a**) Cyclic voltammograms; (**b**) Nyquist plots from EIS of an immunosensor in different stages during the functionalization process: blank, MUA, MUA/MAB17571, MUA/MAB17571/PVA. The insert in (**b**) shows a zoom-in of the Nyquist plot.

The electrochemical properties of the immunosensor were characterized in subsequent stages of the functionalization procedure via CV and EIS measurements using ferro/ferricyanide as a mediator. Figure 4 shows the cyclic voltammograms and Nyquist plots of a blank electrode, the electrode with MUA, the electrode with the immobilized antibody, and the electrode after blocking with PVA. The EIS data were fitted using a Randles circuit (the circuit is shown in Figure S1).

Figure 4a shows an oxidation and reduction peak for the blank, which completely disappears after the formation of the MUA layer. This indicates successful SAM formation, since the negative charge of the carboxylic acid group of MUA shields the electrode surface from ferro/ferricyanide ions. The formation is also confirmed by the data shown in Figure 4b, where the semicircle is dramatically increased in comparison to the blank, indicating an increase in charge transfer resistance ($R_{ct}$) (see Table 2). After the activation of carboxylic acids and covalent immobilization of the antibody, the CV response decreased slightly around the 0.8 V potential. This likely stemmed from the immobilization of antibodies that hindered the electron transport of ferro/ferricyanide ions to the electrode surface. This is also evident from the Nyquist plot in Figure 4b and the $R_{ct}$ value in Table 2, showing an increase in the semicircle size and a doubling of the resistance, respectively. Blocking the electrode surface with PVA resulted in a decrease in current around the 0.8 V potential for CV, an increase in the size of the semicircle in the Nyquist plot, and an increase in the $R_{ct}$. These changes point toward the effective blocking of the surface, where PVA fills in the gaps in the MUA layer, which further obstructs the electron transport of ferro/ferricyanide ions.

### 3.3. Detection and Amplification Strategies

#### 3.3.1. Detection Strategies

The electrochemical detection methods typically used for biosensors are CV, EIS, and chronoamperometry [6,9,43]. Initially, all three methods were evaluated to investigate the feasibility, suitability, and reproducibility of the immunosensors.

**Figure 5:**
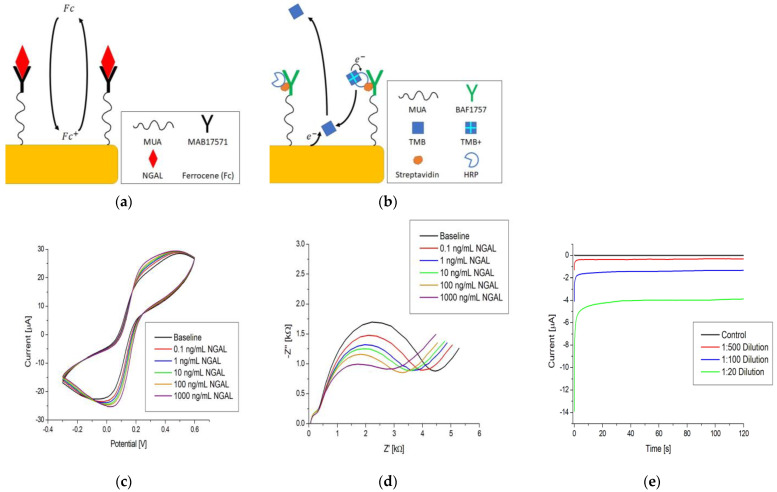

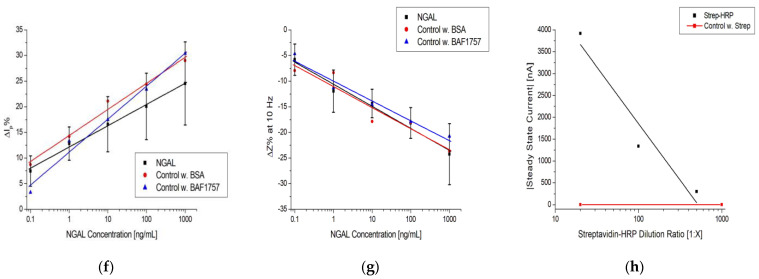
Simplified immunoassay with electrochemical detection via: (**a**) CV and EIS; (**b**) chronoamperometry. Raw data examples of (**c**) CV, (**d**) EIS, and (**e**) chronoamperometry. Log-linear plot of (**f**) $\Delta I_{p}\%$ and (**g**) ΔZ% at 10 Hz vs. NGAL concentration and the steady-state current vs. Strep-HRP dilution ratio. The black, red, and blue lines in (**f**–**h**) are logarithmic fits.

CV and EIS were performed on the immunosensors as described in Section 2.6.2. using ferrocenedimethanol as an electron mediator with antigen concentrations ranging from 0.1 to 1000 ng/mL. A total of five immunosensors were tested. The schematic diagram in Figure 5a illustrates the detection method for CV and EIS with ferrocenedimethanol. Build-up on the surface due to antigen binding to antibodies causes a change in the environment affecting the diffusion and reduction/oxidation process of the mediator, which is observed as changes in the peak current for CV and impedance for EIS. From the voltammograms in Figure 5c, it can be seen that the peak current increases with higher concentrations of antigen, and as shown in the Nyquist plots in Figure 5d, the impedance decreases with higher concentrations. The percent change in the peak current and percent change in the impedance at 10 Hz against the antigen concentration are represented in log-linear plots, Figure 5f,g. Although the current increase and impedance decrease observed upon antigen–antibody binding might seem counterintuitive, these changes have been reported previously and may be attributed to the interaction between the probe and particular surface modifications [44,45,46,47].

The plots in Figure 5f,g show a general trend with the increasing antigen concentration; however, the average coefficients of variation (CV%) are 32.6% for CV and 29.5% for EIS. The signal-to-background ratio (SBR) is within +−0.25 of the baseline, as seen in Table 3. These values indicate that the reproducibility is subpar at best, with little distinction between concentrations and a limited separation between the background and signal. Due to these findings, additional control experiments were performed following the protocol in Section 2.6.2., but instead of using NGAL, we used BSA and BAF1757, which cannot bind to the immobilized antibody. The red and blue datasets in Figure 5f,g are the results of the control experiments, which show the same tendency as that observed when NGAL was used.

Chemiluminescence was subsequently applied to the immunosensors to rule out the possibility that the electrochemical results were influenced by a flawed functionalization procedure, in which no antibodies were immobilized on the SAM. The results confirmed that antibodies were present and immobilized on the immunosensors.

These results indicated that the electrodes were properly functionalized. However, the changes observed in the peak current and impedance were not related to the build-up of antigen on the immunosensor but more likely reflect the nonspecific binding or accumulation of electron mediators or other charged molecules on the working electrode surface, which is a finding that has also been observed for other sensors [16].

Chronoamperometry was performed using TMB as an electron mediator at a 0.0 V potential, as outlined in Section 2.6.1. TMB was validated as an electron mediator and the applied potential of 0.0 V was selected to avoid the electrochemical oxidation of TMB (see the cyclic voltammogram in Figure S2). Figure 5b shows the immunoassay setup and electron transfer pathway for TMB. TMB molecules are oxidized in an enzymatic reaction with HRP and H_2_O_2_ forming TMB^+^. When in proximity to the electrode surface, TMB^+^ can accept an electron and be reduced to TMB electrochemically. This means that the current associated with the electrochemical reduction of TMB^+^ is a measure of the HRP concentration, provided that TMB and H_2_O_2_ are in excess.

The raw data in Figure 5e show a distinction between the dilutions and that after an initial spike, the current stabilizes and reaches a steady state (flattening of the curve) after approximately 100 s. An absolute value of the average current between 100 and 120 s was used as the measurement value. Figure 5h is a log-linear plot of the steady-state current vs. dilution ratio of Strep-HRP, which shows a dependence between the electrochemical signal of TMB and the dilution ratio. It also shows a clear separation between the control measurement (red line), where streptavidin with no HRP attached was used, and the lowest dilution of Strep-HRP at 1:500. Additionally, these results confirm that the electrodes were properly functionalized, as Strep-HRP could not bind to the immunosensor without the presence of biotinylated antibodies, which resulted in no electrochemical signal. The tendencies observed when using chronoamperometry were further substantiated by performing identical experiments on different types of immunosensors. The results are shown in Figure S3, which shows the same trend as Figure 5h.

Comparing the CV, EIS, and chronoamperometry plots in Figure 5f–h, it can be seen that chronoamperometry provided a more robust way to electrochemically quantify an immunoassay. It offered the steepest signal change per concentration unit with a significantly higher SBR, as seen in Table 3. The chronoamperometry detection method was used for experiments thereafter.

#### 3.3.2. Electrochemical Signal Amplification Strategy

**Figure 6:**
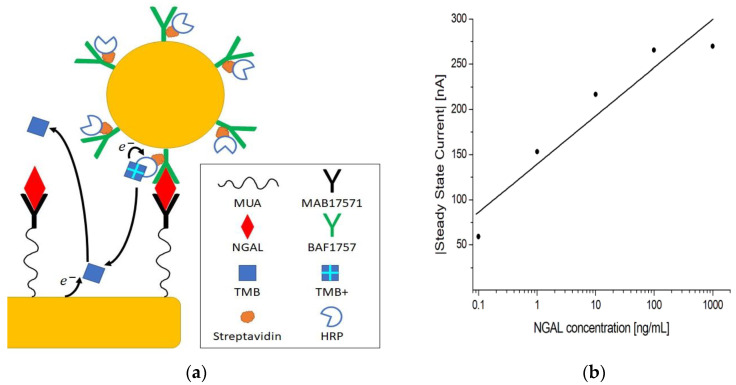
(**a**) Schematic representation of the electrochemical sandwich immunoassay with amplification using AuNP conjugates. (**b**) Log-linear plot of the steady-state current vs. NGAL concentration in PBST using MUA/MAB17571/PVA immunosensors.

The chronoamperometric detection method can be used in combination with immuno-sandwich complexes to render the electrochemical signal dependent on an antigen concentration, as demonstrated in [29,48,49,50,51]. A standard practice for biosensors is to amplify the signal as a means of increasing the sensitivity. To achieve this, different strategies have been suggested, such as nanoparticles, magnetic particles, nanomaterials, enzymes, artificial enzymes, and fluorescence [11,52,53,54,55,56]. In this work, we made use of AuNP conjugates to amplify the steady-state current, as depicted in Figure 6a.

Following the assay procedure outlined in Section 2.6.3, 0.1–1000 ng/mL concentrations of antigen were tested. The log-linear plot in Figure 6b shows that the current increases with increasing concentrations of antigen, with a clear distinction between concentrations. The control measurement without HRP showed almost no current, as seen in Table 4. Additionally, a current was registered when no antigen was present, which means that some nonspecific binding occurred, but this was separable from the measurements at 1 ng/mL and above, as indicated by the SBRs in Table 4. This indicates that when antigen the is present, the signal measured stems from the formation of immuno-sandwich complexes. The amplification mechanism is likely the result of AuNP conjugates carrying multiple HRPs, hence increasing the number of HRPs per unit immuno-sandwich complex [56].

### 3.4. Electrochemical Measurements in Urine Samples

To evaluate the feasibility and potential of the immunosensor for biological fluid applications, it was tested and optimized using complex samples. Urine samples were prepared as outlined in Section 2.6.3. before spiking with antigen.

The blocking method with PVA proved to be insufficient when testing with complex samples, since the substances present (e.g., large proteins) could nonspecifically bind to the immunosensor. To counteract this, the blocking method was changed to the version using Cyst and PEGDA, as described in Section 2.5.3.

To optimize the senor performance, the AuNP conjugate dilution ratio was optimized to increase the current signals. Furthermore, the incubation time and effect of pre-incubation were investigated.

**Figure 7:**
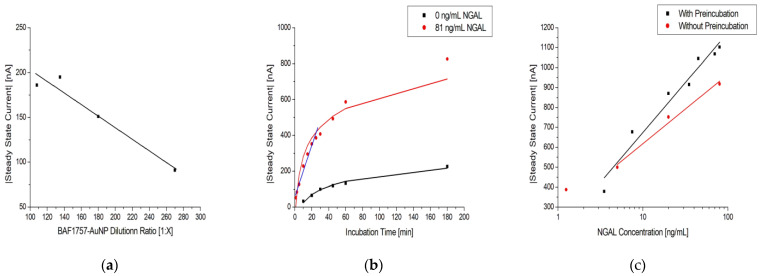
(**a**) Dilution series of BAF1757-AuNP with 1:100 Strep-HRP and 81 ng/mL NGAL in urine samples on MUA/MAB17571/Cyst/PEGDA immunosensors with a linear fit. (**b**) Effect of sample incubation time on MUA/MAB17571/Cyst/PEGDA immunosensors with 0 and 81 ng/mL NGAL in urine samples, colored black and red, respectively. The black and red line are logarithmic fits, while the blue line is linear. (**c**) Log-linear plot of the steady-state current vs. NGAL concentration in urine samples on MUA/MAB17571/Cyst/PEGDA immunosensors with (black) and without (red) the preincubation of NGAL and BAF1757-AuNP-Strep-HRP. The black and red lines are logarithmic fits.

The results in Figure 7a show that when increasing the dilution ratio of AuNP conjugates, the steady-state current decreases in a linear fashion. It also shows a saturation point around the 1:120 dilution ratio mark, indicating that more concentrated dilutions would not bring any benefit for further amplification. Based on this result, the 1:120 dilution ratio was used for the experiments.

Figure 7b shows how the measured steady-state current is affected by the incubation time of a sample using the immunosensor. It shows that there is a continuous increase as incubation time is increased for both the tested sample (red curve) and the control (black curve). However, the largest increase in current occurs within 30 min, as highlighted by the linear fit (blue curve).This indicates that past this point, the increase in the incubation becomes less efficient in generating a higher current. This is also corroborated by the data on the rates of change listed in Table 5, which decrease as the incubation time is increased. The SBRs are also listed in Table 5, being highest at 10 min of incubation time, partially consistent from 30 to 60 min, and lowest at 180 min. The continuous increase in current for the control sample can be attributed to nonspecific binding on the immunosensor. Therefore, to obtain a high current readout, an acceptable SBR, and low nonspecific binding, a 30 min period of sample incubation was selected for the experiments.

The effect of pre-incubation before application to the sensor is shown in Figure 7c. Without pre-incubation, the steady-state current decreased slightly, indicating suboptimal assay formation when the incubation time was decreased. Although it would be possible to skip the pre-incubation, reducing the assay time by 50%, pre-incubation was used to ensure optimal current signals.

### 3.5. Sensing Platform Evaluation

The SPEs used in the study had different morphologies, as shown in Figure 1, Figure 2 and Figure 3, likely attributed to differences in ink composition and fabrication protocols. Therefore, the functionalization protocols generally require optimization for a specific SPE. On the other hand, the exposed surface of thin-film electrodes consists of pure gold, making the functionalization procedure more universally applicable, without dependence on a specific manufacture or electrode type.

**Figure 8:**
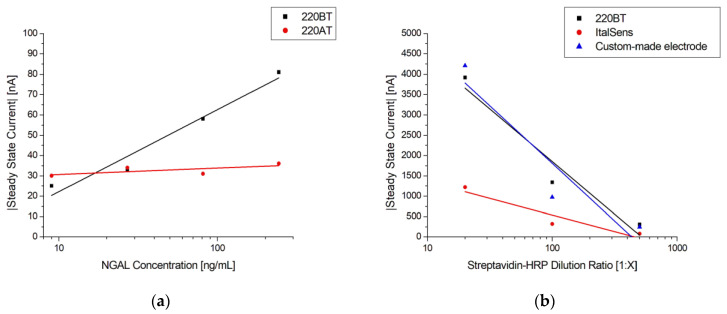
(**a**) Log-linear plot of steady-state current vs. NGAL concentration in PBST with 0.1% BSA using MUA/MAB17571 immunosensors blocked with goat serum on 220BT and 220AT SPEs. The black and red lines are logarithmic fits. (**b**) Log-linear plot of steady-state current vs. Strep-HRP dilution ratio with a logarithmic fit using MUA/BAF1757/Cyst/PEGDA immunosensors on 220BT and ItalSens SPEs and custom-made electrodes.

To investigate the suitability of the developed protocol for different kinds of sensors, three different SPEs and the custom-made thin-film electrodes of similar geometry were used to fabricate immunosensors, employing the method described in Section 2.5. Figure 8a shows the responses of two different SPE types from the same manufacturer, 220AT (red) and 220BT (black), tested with the sandwich immunoassay. The measurements with 220AT did not show a concentration dependence, while the measurements with 220BT did. This indicates that the ink or ink processing in 220AT electrodes is not compatible with the developed protocol, requiring a different functionalization procedure, e.g., layering the MUA on the surface by letting a droplet of MUA evaporate, as suggested by Doldán et al. [57]. On the other hand, the ItalSens SPEs, as well as the custom-made thin-film electrodes, tested with the Strep-HRP assay, as described in Section 2.6.1., exhibited a clear response to the HRP concentration, as shown in Figure 8b. It should be noted that the ItalSens SPEs showed a lower sensitivity to Strep-HRP compared to the custom-made and 220BT electrodes. The 220BT and custom-made electrodes showed almost identical currents, which was somewhat surprising, since the custom-made electrodes should have had a more pristine surface for SAM formation and electrochemical measurements due to the fact that these electrodes were fabricated using high-purity gold (<99%). A possible explanation for this may be the surface area. Since the custom-made electrode is close to planar and the 220BT is coarser with significant variation in height, as shown in Figure 1, Figure 2 and Figure 3, this causes the 220BT to have a larger surface area, which may help to counteract its lower gold purity. A different explanation may be that the functionalization method was not optimal for the custom-made electrode, thereby limiting the performance.

**Figure 9:**
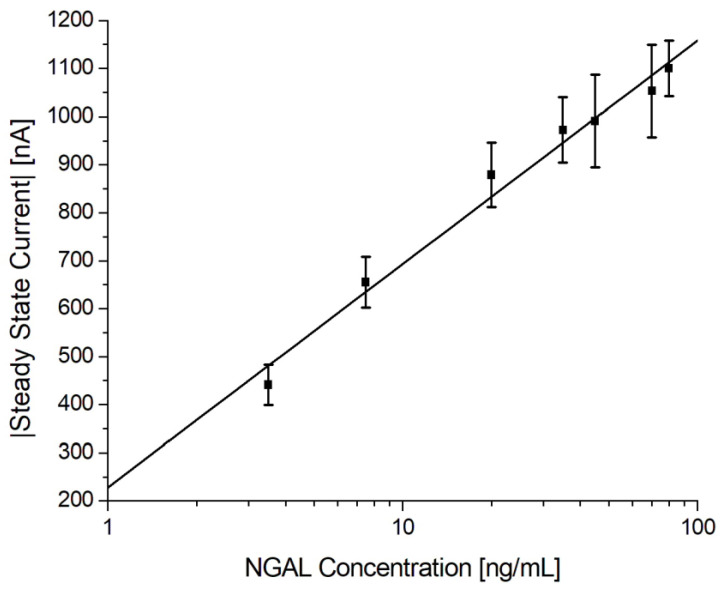
Electrochemical immunoassay for NGAL in spiked urine samples. Log-linear plot of the steady-state current vs. NGAL concentration using MUA/MAB17571/Cyst/PEGDA immunosensors with a logarithmic fit.

To validate the reproducibility and robustness of the electrochemical immunoassay, it was tested using multiple urine samples spiked with different concentrations of antigen, according to the protocol described in Section 2.6.3. The calibration curve of the urine samples can be seen in Figure 9 with the respective data shown in Table 6. It shows a linear relationship between the steady-state current and the logarithmic values of the antigen concentration in the range from 3.5 ng/mL to 80 ng/mL with a theoretical detection limit (LOD) of 1.0 ng/mL, estimated as the mean blank level + 3 × standard deviation of the blank. Although others have reported a similar performance using gold electrodes in a standard three-electrode cell or improved sensitivity using a combination of carbon electrodes and nanomaterials, as presented in Table 7, this work was the first in which such a detection was demonstrated using a commercially available gold SPE for the electrochemical immunosensor in bodily fluids [47,58,59,60,61,62,63,64,65,66].

The logistic regression equation is given as I=A+B×ln⁡(x), where A is 228 and B is 202, with an r^2^ = 0.9822. The results listed in Table 6 show that the inter-assay CV% was 10.5% and 8.0% excluding the blank (n = 72). These results suggest that the electrochemical immunoassay is reproducible and robust.

To demonstrate that the presented electrochemical immunosensor can function as a general platform for immunoassays, tests were performed with VEGF165 and LDL immunoassays using the procedure presented in Section 2.6.3.

**Figure 10:**
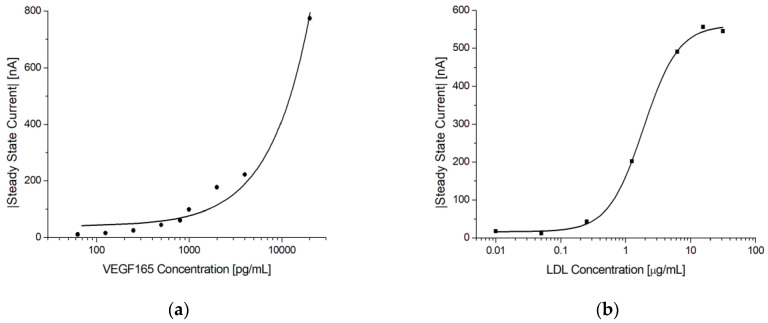
Electrochemical immunoassays for VEGF165 and LDL. (**a**) Log-linear plot of the steady-state current vs. VEGF165 concentration using MUA/AF293/Cyst/PEGDA immunosensors. The line shows a linear fit. (**b**) Log-linear plot of the steady-state current vs. LDL concentration using MUA/LDL20|17/Cyst/PEGDA immunosensors. Note the sigmoidal shape of the response indicating that both the lower and upper sensitivity limits were reached. The solid line shows a 4-parameter logistic fit.

Figure 10 shows the steady-state current vs. antigen concentration for the two immunoassays. In both cases, a concentration dependence was observed. For VEGF165, the current continued to increase at the tested concentrations, indicating that the saturation limit was not reached, whereas for LDL, both the lower and upper limits were observed.

## 4. Conclusions

The morphology and electrochemical behavior of several screen-printed and thin-film electrodes were investigated. It was found that the proposed functionalization strategy was suitable for both the thin-films and most of the SPEs. While the thin-film electrodes offered a well-defined surface chemistry ideal for thiol-based SAM formations, SAMs of sufficient quality could also be formed to successfully immobilize antibodies on most of the SPEs, which was confirmed via CV and EIS measurements. On the other hand, the inherent property of enhanced roughness of the SPEs provided the advantage of a larger surface area. In our experiments, we found that these two factors compensated for each other, leading to similar electrochemical performances for the 220BT and thin-film electrodes.

We evaluated CV, EIS, and chronoamperometry as potential sensing mechanisms for quantitative immunoassays. Chronoamperometry was the most robust technique, offering superior SBRs. We further investigated this detection method using NGAL, VEGF165, and LDL sandwich immunoassays implemented with 220BT electrodes. A different blocking approach using a combination of cysteamine and PEGDA was implemented for measurements with complex samples (urine). Pooled urine samples spiked with 8 different concentrations of NGAL were each measured using 9 different sensors (72 individual immunosensors in total) across 9 days. Based on these data, the linear range was 3.5 ng/mL to 80 ng/mL, with a LOD of 1.0 ng/mL and an inter-assay CV% of 8%, excluding the blank.

In conclusion, we developed an electrochemical immunosensor applicable to immunoassays in bodily fluids, which was found to be robust and reproducible, yielding quantitative readouts within an hour (potentially 30 min). Furthermore, the developed technology can serve as a platform for immunoassays and is suitable for implementation with different electrodes, both SPEs and thin-films. The coupling of this platform with miniature single-chip potentiostats would create a simple and compact device that is fit for a PoC environment.

## Figures and Tables

**Table 1:** Surface roughness analysis of AFM images from Figure 2.

Surface Roughness Analysis of AFM Images	220BT	ItalSens	220AT	MicruX IDE	Metrohm IDE	Custom-Made Electrode
RMS roughness	0.391	0.354	0.215	0.0032	0.0052	0.0081
Roughness coefficient	1.69	1.26	1.11	1.02	1.00	1.03

**Table 2:** Fitted values for charge transfer resistance using a Randles circuit for the EIS data shown in Figure 4b.

Functionalization Process	$R_{ct}\;[k\Omega ]$
Blank	0.034
MUA	676
MUA/MAB17571	1390
MUA/MAB17571/PVA	2038

**Table 3:** Signal-to-background ratio for each tested concentration in Figure 5f–h when using CV, EIS, or chronoamperometry as the detection method.

Detection Method	Testing Solution	Concentration	SBR
CV	NGAL	0.1	1.07
		1	1.13
		10	1.17
		100	1.20
		1000	1.25
EIS	NGAL	0.1	0.94
		1	0.88
		10	0.86
		100	0.82
		1000	0.76
Chronoamperometry	Strep-HRP	1:500	61
		1:100	268
		1:20	784

**Table 4:** Tested NGAL concentrations and steady-state currents from Figure 6b with signal-to-background ratios.

NGAL Concentration (ng/mL)	Current (nA)	SBR
Control ^1^	4	-
0	53	-
0.1	59	1.11
1	153	2.89
10	217	4.09
100	265	5.00
1000	270	5.09

^1^ Using BAF1757-Strep instead of BAF1757-AuNP-Strep-HRP.

**Table 5:** The rate of change for immunosensors with 81 ng/mL NGAL and the signal-to-background ratio between 81 and 0 ng/mL NGAL at various incubation times from Figure 6b.

Incubation Time (min)	Rate of Change (nA/min)	SBR
10	22.8	6.91
20	17.5	5.40
30	13.6	4.07
45	10.9	4.13
60	9.8	4.44
180	4.6	3.65

**Table 6:** Tested NGAL concentrations in spiked urine samples and steady-state currents, including the standard deviations and number of repetitions of the NGAL electrochemical immunoassay. LOD = 1.0 ng/mL, average inter-assay CV% = 10.5% (8.0% excluding blank). A total of 72 immunosensors were tested.

NGAL Concentration (ng/mL)	Current (nA)	Standard Deviation (nA)	CV%	# of Repetitions
0	124	35	27.9	9
3.5	441	42	9.4	9
7.5	655	53	8.1	9
20	879	67	7.6	9
35	972	68	7.0	9
45	991	96	9.7	9
70	1053	96	9.1	9
80	1100	58	5.2	9

**Table 7:** Comparison of LOD, linear range, and total number of tests for the calibration curve between NGAL biosensors using different materials.

Material	LOD	Linear Range	# of Tests	References
Gold electrode/AuNPs/PAMMAM immunosensor	1 ng/mL	50–250 ng/mL	-	[58]
Graphite SPE modified with a graphene oxide composite and AuNP aptasensor	0.3 ng/mL	1–1000 ng/mL	33	[59]
Graphene/polyaniline-modified carbon SPE immunosensor	21.1 ng/mL	50–500 ng/mL	32	[60]
Casein-modified carbon SPE immunosensor	97 pg/mL	0.15–2 ng/mL	42	[61]
Carbon fiber immunosensor with boron carbon nitride nanosheets	0.37 pg/mL	0.001–10 ng/mL	25	[62]
Graphene nickel foam/AuNPs/SAM/immunosensor	42 pg/mL	0.05–210 ng/ml	-	[47]
Gold nanourchins and carbon nanohorns modified carbon SPE aptasensor	10 fg/mL	0.1–100 pg/mL	-	[63]
Gold electrode aptasensor	4.45 ng/mL	25–150 ng/mL	21	[64]
Nickel oxide nanoparticles modified cerium copper oxide thin film on silicon	4.23 ng/mL	25–450 ng/mL	-	[65]
Gold-coated quartz electrode modified with peptide imprinted film	70 ng/mL	1–300 µg/mL	-	[66]
Gold SPE/MUA/immunosensor	1 ng/mL	1–80 ng/mL	72	This work

## Data Availability

The data presented in this study are available on request from the corresponding author.

## Supplementary Materials

The following supporting information can be downloaded at: https://www.mdpi.com/article/10.3390/bios13050519/s1. Figure S1: Randles circuit. Figure S2: Cyclic voltammogram of 1-Step Turbo TMB-ELISA Substrate Solution on a blank 220BT electrode to identify the potential region where no electrochemical oxidation of TMB occurs. Figure S3: Additional experiments with streptavidin-HRP dilution series to validate the trend seen for the chronoamperometry sensing mechanism. Table S1: The steady-state currents from the additional experiments with streptavidin-HRP dilution series.
