# Supplementary material for: Amperometric Biosensor for Quantitative Measurement Using Sandwich Immunoassays
Source: Biosensors (Basel). 2023 May 5;13(5):519. doi: 10.3390/bios13050519 (PMC10216821; doi:10.3390/bios13050519)
Supplement: Supplementary file 1 [file biosensors-13-00519-s001.zip › biosensors-2352741-supplementary.pdf]

Supporting Information:

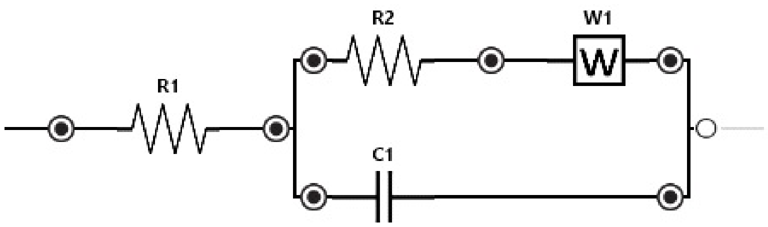

Figure S1. Randles circuit used for fitting electrochemical characterization EIS data.

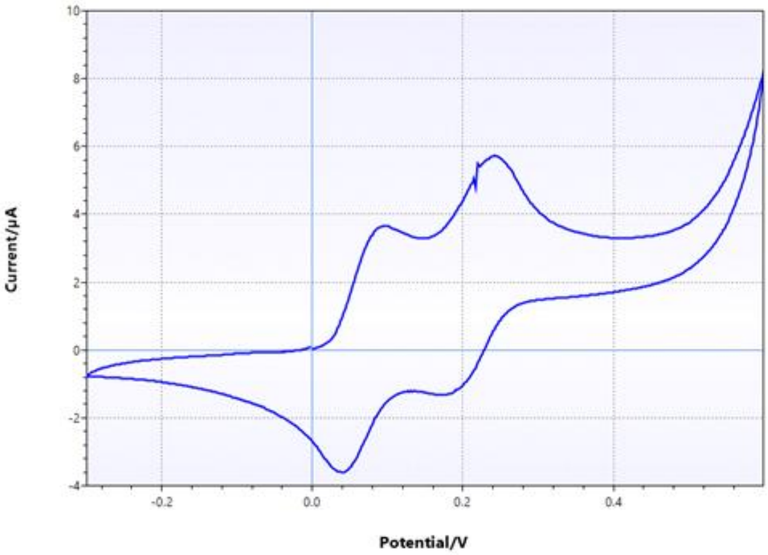

Figure S2. Cyclic voltammogram of 1-Step Turbo TMB-ELISA Substrate Solution on a blank 220BT electrode.

Figure S2 shows the cyclic voltammetry data of the 1-Step Turbo TMB-ELISA Substrate Solution on a blank 220BT electrode. As seen in the figure TMB is an electrochemical active molecule with 2 oxidation and reduction peaks, 1 pair is situated around 0.06 V and the other around 0.2 V. The first pair represents TMB/TMB^{+} while the second is TMB^{+}/TMB^{++}. To use the TMB molecules as electron mediators and link them to a specific concentration it is critical to ensure no electrochemical oxidation takes place and only the enzymatic reaction with HRP, H2O2 and TMB is contributing to the electrochemical signal. Therefore, the potential used in chronoamperometry needs to be below the formal potential of the TMB/TMB^{+} redox reaction. As it can be seen from Figure S2 a potential of 0.0 V satisfies this requirement.

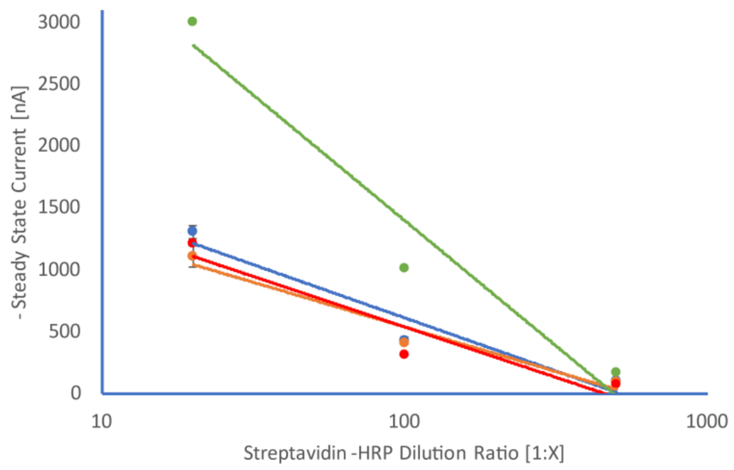

Figure S3. Log-linear plot of steady state current vs. Strep-HRP dilution ratio using different immunosensors. The blue, orange, red and green lines are logarithmic fits using MUA/BAF1757/PVA, MUA/BAM/PVA, MUA/BAF1757/Cyst/PEGDA and MUA/BAF293/Cyst/PEGDA immunosensors respectively. All sensors used here were fabricated using 220BT electrodes expect for MUA/BAF1757/Cyst/PEGDA (red) which used ItalSens.

Figure S3 shows the results from additional experiments with Strep-HRP dilution series in a log-linear plot. As shown immunosensors with three different immobilized antibodies, two different blocking methods, and two electrode types show a dependence for the Strep-HRP dilution ratio when measured using chronoamperometry. The MUA/BAF1757/PVA and MUA/BAM/PVA immunosensors have two replications for each concentration which gave a mean CV% of 6.6% and 5.3%, respectively.

Table S1. Steady state current for tested dilutions across different immunosensors.

Electrode	Immunosensor	Concentration	Current [nA]
220BT	MUA/BAF1757/PVA	1:500	104
		1:100	427
		1:20	1307
	MUA/BAM/PVA	1:500	97
		1:100	413
		1:20	1107
	MUA/BAF293/Cyst/PEGDA	1:500	173
		1:100	1016
		1:20	3009
ItalSens	MUA/BAF1757/Cyst/PEGDA	1:500	78
		1:100	319
		1:20	1220
